# The Association of Pleurisy with Appendicitis

**Published:** 1901-11-30

**Authors:** 


### THE ASSOCIATION OF PLEURISY WITH APPENDICITIS.

Speaking before the Hunterian Society on the subject of the chest complications in abdominal disease, Dr. Mitchell Bruce referred to a matter which is of considerable importance, namely, the occurrence of acute pleurisy in the course of, or as a consequence of, appendicitis. By far the commonest form of peritonitis found in association with acute pleurisy and other complications within the chest is perityphlitis originating in appendicitis. The chest complication does not by any means always come on during the active stage of the abdominal disease, and at first sight it appears remarkable that, as in a case which was related, whilst the right lung and pleura were actively involved in what must be regarded as a process of local infection, the source of the infection, namely, the appendicitis, should have been perfectly quiescent. Of the fact there can be no doubt; indeed the chest may be invaded in cases of perityphlitis even after successful surgical intervention, including removal of the appendix, and even when on most careful examination nothing can be discovered to suggest the continuance of any inflammatory condition about the site of the original disease. The gravity and extent of the chest complication may vary greatly, sometimes being a mere evanescent pleurisy, quickly passing away, while in other cases the secondary disease may be of the most serious nature. What is very interesting is that in some cases evacuation of the intraperitoneal abscess is sufficient to relieve both conditions. On the other hand, an empyema so arising may extend its course over months and may gravely compromise the prospects of recovery, or right pleuro-pneumonia may be found lying over a subdiaphragmatic abscess which connects it with the diseased area round the appendix. In fact, not only may appendicitis set up the gravest forms of chest complications by what one may presume to be a process of local extension, but infection may spread from the appendix by way of the portal vein and the liver, and ultimately set up disease in the chest—septic endocarditis, for example, by a process of pyæmia. The whole subject is one of very considerable clinical importance. However well a case of appendicitis may seem to be going on, the significance and possible relationship of any intercurrent chest disorder must be remembered; while, on the other hand, in seeking for the cause of a pleurisy, a subdiaphragmatic abscess, or an empyema, one must not overlook the possibility of its having arisen from some perhaps forgotten or overlooked attack of appendicitis.

